# Aortitis as a Harbinger of Occult Malignancy

**DOI:** 10.1155/2019/8385630

**Published:** 2019-02-26

**Authors:** Erik W. O'Connell, Jennifer Reams, Alfred E. Denio

**Affiliations:** Division of Rheumatology MC 2152, Geisinger Medical Center, 100 N Academy Ave., Danville, PA 17822, USA

## Abstract

Noninfectious aortitis can be associated with an occult malignancy. Although glucocorticosteroids are often used, there is no clear evidence-based therapy and there is little consensus regarding treatment. Identifying and treating an underlying trigger is the most efficacious therapy. We present an unusual case initially concerning acute bacterial endocarditis of the native mitral valve; however, aseptic ischemic valvitis manifested on pathology. Concurrent aortitis was diagnosed with angiography. Occult colon adenocarcinoma was discovered during diagnostic abdominal imaging. Treatment of this underlying malignancy was associated with dynamic mitigation of inflammation affecting the entire aorta and a reduction in associated symptoms. This is an unusual case of a paraneoplastic secondary large vessel vasculitis involving the entire aorta, a diagnosis of exclusion but a consideration that can have dramatic impact on both morbidity and mortality.

## 1. Introduction

Aortitis is a term that describes either an infectious or noninfectious disease process that produces inflammation of the aorta. Diagnosis is often established radiographically or, more commonly, based on tissue diagnosis following the resection of an aortic segment due to aneurysm or dissection. When radiographic diagnosis is established, it is typically based on CT, MRI, or PET scan findings [1]. Infectious causes of aortitis should be excluded. The most common cause of noninfectious aortitis is giant cell arteritis. Other potential etiologies would include Takayasu's arteritis, rheumatoid arthritis, systemic lupus erythematosus, Cogan's syndrome, Behcet's disease, ankylosing spondylitis, and IgG4-related disease [2]. When systemic etiologies have been excluded, a patient is classified as having idiopathic aortitis. Aortoplasty has been reported as a trigger of this pathology. Idiopathic aortitis can be an early manifestation of systemic vasculitis [3]. Managing this disease can be challenging. Unless a trigger can be identified, immunosuppression is often used. Patients with this pathology are at risk for aneurysm and dissection [4].

Aortitis can be divided into four categories: granulomatous (GCA, mycobacterial, sarcoid, GPA, rheumatoid, and Takayasu), lymphoplasmacytic (IgG4, syphilis, SLE, and ankylosing spondylitis), suppurative (typically infection with pseudomonas, salmonella, or Gram-positive cocci), and mixed inflammation (Cogan's, relapsing polychondritis, and Behcet's disease). The most common is granulomatous [5].

## 2. Case Presentation

A 47-year-old school superintendent presented to his primary care physician with nonproductive cough, persistent fever, and night sweats after two weeks of progressive insidious symptoms. He had no history of arthritis, arthralgia, myalgia, or rash. He did have many sick close contacts at work. He denied illicit drug use.

On exam, there was a prominent axillary holosystolic murmur and he was directed to the emergency room. He was admitted with concern for endocarditis with bacterial systemic infection. ESR was 104 mm/hr and CRP 69 mg/L. Blood cultures were drawn, and empiric antibiotics were started. Transthoracic echocardiogram was performed and revealed a flail mitral valve.

Cardiothoracic surgery attempted repair but ultimately replaced the valve, sending tissue for pathology which revealed aseptic fibrinous vegetation with ischemic valvitis. All cultures were negative.

Aortic inflammation was incidentally noted on screening imaging of the chest, abdomen, and pelvis which also identified a colon mass. Inflammation was limited to the aorta based on this imaging with no concurrent involvement of either the subclavian arteries or alternate arterial branches. He was treated with 1 mg/kg/day of prednisone with long taper. At one month, moderately differentiated invasive colon adenocarcinoma was excised and pathology revealed transmural disease without node involvement and clean margins. Symptoms improved. Aortic inflammation decreased.

**Figure 1:**
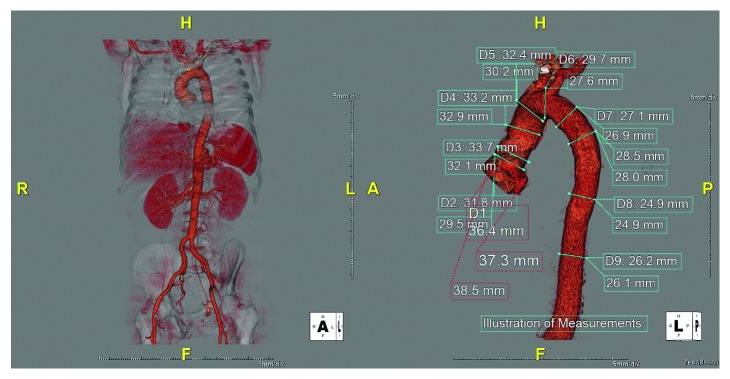
CT angiogram with 3D reconstruction demonstrating aortitis without involvement of adjacent branches.

Two months later, he had a similar presentation. This time, however, he had chest pain: CXR normal; EKG normal; V/Q scan low probability; TTE without pericardial disease and nonpathologic bioprosthetic valve in good position; ESR >100; and CRP 206. CT angiography of the chest, abdomen, and pelvis revealed recurrence of the colon mass and inflammation of the entire aorta. ANA screen, cultures, immunotesting for potential pathogens, and ANCA were all negative. Angiography was performed (Figure 1) and revealed large vessel vasculitis affecting the entire aorta. Pulse-dosed steroids were again initiated with slow prednisone taper.

After surgical intervention successfully excised the neoplasm, our patient had improvement in symptoms and a decrease in aortic inflammation. Consideration was given for steroid-spearing treatment with either rituximab or tocilizumab. Unfortunately, rituximab was denied by the patient's insurance and was not further pursued. To date, significant improvement without recurrence has been shown with a steady steroid taper and therefore tocilizumab has also not been further pursued. Contemplation for its future use remains an option in the event of an unsuccessful steroid weaning. The patient is being monitored regularly via self-reported symptomology as well as recurrent lab work to include CBC with differentiation to screen for anemia and inflammatory markers. The last known inflammatory markers were within normal range.

## 3. Discussion

Secondary large vessel vasculitis is typically diagnosed with supportive tissue evaluation by a pathologist following aortic aneurysm or dissection repair. Alternatively, radiographic identification can be made using enhanced computed tomography, magnetic resonance, or positron electron tomography. Our patient presented with features concerning bacterial endocarditis, meeting modified Duke criteria. Severe mitral regurgitation was compelling for valvuloplasty which revealed aseptic ischemic valvitis. It was important to exclude infectious etiology, especially fungal and bacterial pathogens. Literature review does suggest that aortitis can be associated with systemic vasculitis. Serologic screening for common rheumatologic disease is recommended. Following the exclusion of giant cell arteritis and Takayasu arteritis, this patient became a challenging diagnostic and therapeutic dilemma. With the incidental finding of a colon adenocarcinoma and its subsequent treatment, the patient had marked improvement in symptoms of vasculitis. High-dosed steroids did not have much clinical impact on his disease activity; however, excision of neoplasm correlated with improved symptoms and aortic inflammation. Recurrence of colon adenocarcinoma was heralded by symptoms of aortitis. Again, subsequent excision corresponded to improvement in degree of aortic inflammation and ultimately a resolution of symptoms. Paraneoplastic aortitis is an important consideration that treatment of the underlying malignancy can result in decreased degree of large vessel inflammation, especially germane to patients with symptomatic disease recalcitrant to immunosuppression. After exhaustive literature review, this appears to be the first reported case of paraneoplastic panaortitis.
